# Effect of early calf-hood nutrition on the transcriptomic profile of subcutaneous adipose tissue in Holstein-Friesian bulls

**DOI:** 10.1186/s12864-018-4681-2

**Published:** 2018-04-24

**Authors:** Anne-Marie English, Sineád M. Waters, Paul Cormican, Colin J. Byrne, Seán Fair, David A. Kenny

**Affiliations:** 1 Animal and Bioscience Research Department, Teagasc Grange, Dunsany, Co. Meath, C15 PW93 Ireland; 2 Laboratory of Animal Reproduction, Department of Biological Sciences, School of Natural Sciences, Faculty of Science and Engineering, University of Limerick, Limerick, V94 T9PX Ireland; 3 School of Agriculture and Food Science, University College Dublin, Belfield, Dublin 4, Dublin, D04 N2E5 Ireland

**Keywords:** Gene expression, Reproduction, Adipogenesis, Amino acids, Mitochondrial dysfunction, Puberty

## Abstract

**Background:**

Adipose tissue is a major endocrine organ and is thought to play a central role in the metabolic control of reproductive function in cattle. Plane of nutrition during early life has been shown to influence the timing of puberty in both male and female cattle, though the exact biological mechanisms involved are currently unknown. The aim of this study was to investigate the effect of early calf-hood nutrition on the transcriptomic profile of subcutaneous adipose tissue in Holstein-Friesian bulls to identify possible downstream effects on reproductive physiology.

**Results:**

Holstein-Friesian bull calves with a mean (±S.D.) age and bodyweight of 19 (±8.2) days and 47.5 (±5.3) kg, respectively, were assigned to either a high (*n* = 10) or low (*n* = 10) plane of nutrition. Calves were fed in order to achieve an overall growth rate of 1.08 and 0.57 kg/day for the high and low plane of nutrition treatments, respectively. At 126 days of age, the bulls were euthanized, subcutaneous adipose tissue samples were harvested and RNAseq analysis was performed. There were 674 genes differentially expressed in adipose tissue of calves on the low compared with the high plane of nutrition (*P* < 0.05; FDR < 0.05; fold change > 2.0). High plane of nutrition positively altered the expression of genes across an array of putative biological processes but the most dominant cellular processes affected were cellular energy production and branched chain amino acid degradation. A high plane of nutrition caused upregulation of genes such as leptin (*LEP*) and adiponectin (*ADIPOQ*), which are known to directly affect reproductive function.

**Conclusions:**

These results provide an insight into the effect of augmenting the plane of nutrition of Holstein-Friesian bull calves in the prepubertal period on the transcriptome of adipose tissue.

**Electronic supplementary material:**

The online version of this article (10.1186/s12864-018-4681-2) contains supplementary material, which is available to authorized users.

## Background

The advent of genomic selection in the dairy industry has resulted in the identification of sires for use in artificial insemination within weeks of birth and semen from these young bulls is in high demand once they reach puberty. Studies have shown that plane of nutrition in early calf-hood plays a critical role in the timing of the onset of puberty in bulls [1–3]. Recently, our research group has demonstrated that a high plane of nutrition prior to, but not after 6 months of age can accelerate puberty in Holstein-Friesian bulls by approximately 1 month [3]. This has been associated with the advancement of a transient rise in systemic concentrations of luteinising hormone (LH), which normally occurs between approximately 8 and 20 weeks of age [4]. There are many organs implicated in signalling nutritional status to the hypothalamus and anterior pituitary to stimulate gonadotropin releasing hormone (GnRH) and LH secretion, respectively, with white adipose tissue (WAT) key amongst these.

White adipose tissue has traditionally been known for its role in energy storage and release when energy expenditure is greater than energy intake; however, WAT is metabolically active and can therefore influence many bodily systems. The function of WAT in metabolic and reproductive processes is complex but it has been postulated that there is cross-talk between adipokines and the hypothalamic-pituitary-testicular (HPT) axis. Adipogenesis and fat development are influenced by genetics, diet, body depot location and age [5, 6]. There is evidence that offering calves high starch diet during early calfhood induces precocious adipogenesis and lipid filling [7]. It is postulated that the period of potential for stem cells to differentiate into adipocytes is very limited during early life development [8]. Adipose hormones such as leptin production increases proportionally in line with an increase in body fat [9]; evidently then, it is a signal of energy sufficiency [10]. It has been reported that subcutaneous adipose tissue has a higher concentration of both leptin and adiponectin protein than that of visceral adipose [11]. Leptin signals various bodily systems, including the HPT axis, of metabolic energy status and reserves [12]. Studies have shown that while there is an absence of leptin receptors on some key reproductively related cells such as GnRH neurons [13]; the effects of leptin on GnRH release have been shown to be mediated by kisspeptin in some species [14]. Adiponectin, another adipokine hormone, has been shown to have its receptors, ADIPOR1 and ADIPOR2, expressed in the anterior pituitary and the arcuate nucleus in the hypothalamus of cows [15]; therefore, adiponectin employs two potential pathways to signal to the HPT axis.

It has been reported in children that consistent excessive energy intake during early life development can contribute to precocious puberty [16]. In bull calves of dairy breeds early pubertal onset is advantageous to facilitate early semen collection. The exact metabolic mechanisms are unknown; hence this study aimed to examine the effect of early life plane of nutrition on global gene expression profiles of adipose tissue in Holstein-Friesian bulls within the particular context of potential latent implications for HPT function. Much of the published work to-date on this topic has focused on the effect of BCS (body condition score) on adipose tissue hormone signalling in post pubertal animals and there is a dearth of information on the role of adipose tissue in stimulating the HPT axis and its potential as a key regulator of the timing of puberty onset in cattle.

## Methods

All procedures involving animals were approved by the Teagasc Animal Ethics Committee (TAEC30/2013) and were licensed by the Health Products Regulatory Authority, Ireland in accordance with the European Union Directive 2010/36/EU.

### Animal model

This experiment was conducted as part of a larger study designed to examine the effect of early calf-hood nutrition on the physiological control of the HPT axis. Holstein-Friesian bull calves (*n* = 20) with a mean (±S.D.) age and bodyweight of 19 (±8.2) days and 47.5 (±5.3) kg, were purchased from commercial dairy farms and blocked based on sire, initial weight, farm of origin and age and assigned to either a high (*n* = 10) or low (*n* = 10) plane of nutrition. Calves were individually fed milk replacer and concentrates using an electronic feeding system (Forster-Tecknik Vario, Engen, Germany). The chemical analysis of the milk replacer and the concentrate offered during the trial is outlined in Tables 1 and 2. After 5 days of acclimatisation, the high treatment were offered 1200 g of milk replacer in 8 L of water daily, together with concentrate ad libitum. Animals in the low treatment group were allocated 500 g of milk replacer in 4 L of water and a further allocation of a maximum of 1 kg of concentrate daily. All calves were weaned when consuming a minimum of 1 kg of concentrate for 3 consecutive days, at a mean age (±S.D.) of 82 (±3.9) days. Following weaning, the high treatment group was offered ad libitum concentrates, while the low treatment group received 1 kg of concentrate daily. All calves had individual daily access to approximately 0.5 kg of straw as well as ad libitum access to fresh water throughout the trial period. Animals were weighed weekly over 16 weeks of the trial.

**Table 1:** Chemical composition of milk replacer

	Milk Replacer
Chemical composition (g/kg)	
ADF	12.0 ± 1.98
Crude ash	65.7 ± 2.22
CP	216.3 ± 1.24
DM (%)	96.7 ± 0.15
NDF	5.1 ± 1.00
Oil B	235.0 ± 44.10

*ADF* Acid Detergent Fibre, *CP* Crude Protein, *DM* Dry matter, *NDF* Neutral Detergent Fibre, *Oil B* Acid Hydrolysis

**Table 2:** Diet and chemical composition of concentrate diet offered

	Concentrate
Diet composition (%)	
Rolled Barley	26.5
Soya bean meal	25
Maize	15
Beet pulp	12.5
Soya hulls	12.5
Molasses	5
Mineral and vitamins	2.5^a^
Vegetable oil	1
Chemical composition (g/kg)	
ADF	103.1 ± 6.76
Crude ash	68.8 ± 0.91
CP	167.9 ± 1.86
DM (%)	88.9 ± 0.66
NDF	204.3 ± 18.2
Oil B^b^	30.8 ± 0.72

^a^Mineral and vitamin composition: vitamin A (10 mIU/kg), vitamin D_3_, (2 mIU/kg), vitamin E (40 mg/kg), iodine (8 mg/kg), cobalt (40 mg/kg), copper (88 mg/kg), manganese (81 mg/kg), zinc (139 mg/kg) and selenium (11 mg/kg). ^b^Oil B = Acid hydrolysis

### Tissue collection

The calves were euthanized at a mean age (±S.D.) of 126 (±1.1) days, using an intravenous overdose of sodium pentobarbitone. Death was confirmed by lack of ocular response. Subcutaneous adipose samples were obtained from the flank of the carcass following slaughter. The epidermis and dermis layers were cut to reveal the subcutaneous adipose. The tissue samples were washed with Dulbecco’s PBS and any blood vessels were removed. The tissue was subsampled and the subsamples were either (i) snap-frozen in liquid nitrogen and subsequently stored at − 80 °C for long term storage pending further analysis or (ii) fixed in 10% neutral buffered formalin (Sigma-Aldrich, Wicklow, Ireland) and embedded in paraffin in accordance to the standard procedures. Sections (5 μm thick) were dehydrated in ascending concentrations grades of alcohol, followed by clearing with xylene and were stained for gross anatomy using haematoxylin and eosin (H&E) stain.

### Adipocyte cell number and diameter

Adipocyte cell number and cell size were analysed using Aperio ImageScope (v12; Leica Biosystems, Wetzlar, Germany) from the H&E stain slides. Five square sections (0.25 mm^2^) were electronically generated and randomly assigned on each slide and every adipocyte within these square sections was counted manually. The diameter of 200 adipocytes per animal, selected at random, within these square sections was measured at the widest point for each cell, using the ruler tool from the ImageScope software. The evaluator was blind to the treatments.

### Statistical analysis on adipocyte cell size and number

Data for adipocyte cell size and number and bodyweight were analysed using the procedures of Statistical Analysis Software (SAS version 9.3, Cary, NC, USA) and were tested for normality (UNIVARIATE procedure). Residuals for this data were found to be normally distributed and were statistically analysed using ANOVA (MIXED procedure). Animal was the experimental unit and was included as a random effect. Sampling time (week of weight recording) was included in the statistical models as a repeated measure for weights. All results are presented as mean ± s.e.m.

### RNA isolation and purification

Total RNA was extracted using RNeasy Lipid Tissue Mini kit from 100 mg of subcutaneous adipose (Qiagen, Manchester, UK). RNA was purified using the RNA Clean & Concentrator kit (Zymo Research, Irvine, CA, USA). The quantity of the RNA isolated was determined by measuring the absorbance at 260 nm using a Nanodrop spectrophotometer ND-1000 (Nanodrop Technologies, Wilmington, DE, USA). RNA quality was assessed on the Agilent Bioanalyzer 2100 (Agilent Technologies Ireland Ltd., Dublin, Ireland) using the RNA 6000 Nano Lab Chip kit (Agilent Technologies Ireland Ltd., Dublin, Ireland). Samples had an RNA Integrity Number (RIN) of mean (±SD) of 7.6 (±0.77).

### RNAseq library preparation and sequencing

Twenty cDNA libraries were prepared from high quality RNA using an Illumina TruSeq RNA Sample Preparation kit v2 following the manufacturer’s instructions (Illumina, San Diego, CA, USA). For each sample, 1 μg of total RNA was used for cDNA preparation. All libraries were validated on the Agilent Bioanalyzer 2100 using the DNA 1000 Nano Lab Chip kit (Agilent Technologies Ireland Ltd., Dublin, Ireland). Individual RNAseq libraries were pooled (10 libraries per lane) based on their respective sample-specific-6 bp adaptors and sequenced at 100 bp/sequence single-end reads using an Illumina HiSeq 2500 sequencer.

### RNAseq data analyses

Raw sequence reads were checked for quality using FASTQC software (version 0.10.0) (http://www.bioinformatics.babraham.ac.uk/projects/fastqc/). Input reads were then aligned to the bovine reference genome (UMD3.1) using Spliced Transcripts Alignment to a Reference (STAR) aligner. Reads were only aligned if they contained fewer than two mismatches with the reference genome and uniquely mapped to the reference genome. The software package HTSeq (v0.5.4p5) was used to calculate the number of sequence reads overlapping all protein coding genes from the ENSEMBLv74 annotation of the bovine genome. The number of read counts mapping to each annotated gene from HTSeq was then collated into a single file and used for calculation of subsequent differential gene expression. Only uniquely mapped reads were used for subsequent differential gene expression analysis.. Reads were filtered out if they contained fewer than five counts per million in nine samples out of the total sample number from subsequent analysis. The R (v 3.01) Bioconductor package EdgeR (v3.2.4), was applied to identify statistically significant differentially expressed genes (DEGs).

### Pathway analysis

Biological pathways were identified using GOSeq software (v1.14.0) [17] and Kyoto Encyclopaedia of Genes and Genomes (KEGG) pathway annotations [18] were used to identify biological pathways that were over-expressed among DEGs. GOSeq is an application for executing gene ontology analysis on RNAseq data while accounting for biases [17]. The online tool BioMart (www.ensembl.org/biomart/martview) was used to convert the bovine gene IDs, extracted from GOSeq, into human orthologs. These human orthologs were inputted into KEGG (http://www.genome.jp/kegg/pathway.html). To examine the molecular functions and biological pathways, the RNAseq data was also analysed using Ingenuity Pathway Analysis (IPA) (Ingenuity Systems, Redwood City, CA; https://www.qiagenbioinformatics.com/products/ingenuity-pathway-analysis/).

## Results

### Animal performance and adipose tissue histology

**Fig. 1:**
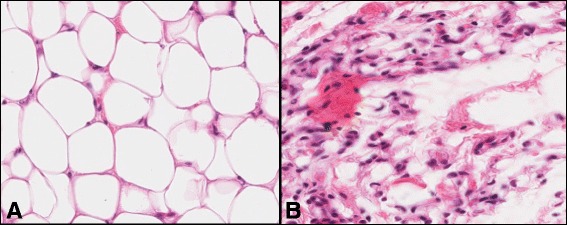
Light micrograph of subcutaneous adipose tissue stained with haematoxylin and eosin. A; High plane of nutrition had large areas of adipocytes (400×), B; Low plane of nutrition had small pockets of adipocytes (400×)

Average daily bodyweight gain from the start to the end of the study was 1.08 ± 0.03 kg and 0.57 ± 0.03 kg for high and low plane of nutrition groups, respectively and this translated into a bodyweight difference of 53.8 kg at slaughter between the bulls of the high compared to the low treatment group (160.9 ± 3.98 kg versus 107.1 ± 3.19 kg, respectively; *P* < 0.001). Adipocyte cell number (291 ± 47.2 cells/1.25 mm^2^ versus 7.5 ± 4.0 cells/1.25 mm^2^, *P* < 0.001; Fig. 1) and cell diameter (36.9 ± 2.89 μm versus 3.7 ± 1.94 μm, *P* < 0.001) were both greater in the high compared with the low plane of nutrition animals, respectively.

### Differential gene expression

**Fig. 2:**
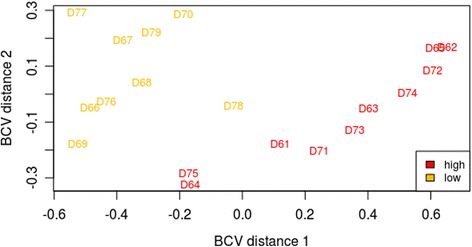
Multidimensional scaling plot which shows the measured similarity of the samples in 2-dimensions. The samples labelled in yellow are the subcutaneous adipose tissue of Holstein-Friesian dairy bulls fed on a low plane of nutrition and slaughtered at 18 weeks of age and those labelled in red are the subcutaneous adipose tissue of Holstein-Friesian dairy bulls fed on a high plane of nutrition and slaughtered at 18 weeks of age. BCV = Biological Coefficient of Variation

The mean ± SD number of raw reads per sample was; 18,670,029 ± 4,093,759. Approximately 89% of the reads were uniquely mapped to the reference bovine genome after alignment. A multi-dimensional scaling (MDS) plot was created in Edge-R which estimated the degree of similarity between samples from the high compared to the low nutritional treatments. The MDS plot showed a separation between the two treatments (Fig. 2). There were 745 differentially expressed genes (DEG) between the two nutritional treatments (*P* < 0.05; False Discovery Rate < 0.05; fold change > 2.0). One hundred and sixty four genes had increased expression, while 581 had decreased expression in the low plane of nutrition compared to the high plane of nutrition. The RNAseq unprocessed data have been deposited in NCBI’s Gene Expression Omnibus and area accessible via GEO series accession number GSE97674.

### Pathway analysis

There were 678 DEG from the original 745 that mapped successfully to a molecular/biological pathway using IPA and these DEG were analysed and allocated to a biological function within IPA. Information on the effect of nutritional treatment on the molecular and cellular functions and on the biochemical pathways affected are presented in Additional files 1 and 2, respectively. Results of KEGG pathway analysis using GOSeq (FDR < 0.05) indicated that 23 KEGG pathways were over-represented among DEG in the low treatment compared to the high treatment at 18 weeks of age (Table 3). These enriched pathways included metabolic pathways, valine, leucine and isoleucine degradation and citrate cycle. After analysis using IPA, the following pathways and processes were identified as the most influenced by the differential nutritional regimen at the level of subcutaneous adipose tissue: energy production via mitochondrial dysfunction (*P* < 0.0001), oxidative phosphorylation (*P* < 0.0001), and tricarboxylic acid (TCA) cycle II (*P* < 0.0001), amino acid metabolism via valine and isoleucine metabolism (*P* < 0.0001). The directionality of the DEG relating to mitochondrial dysfunction, oxidative phosphorylation, TCA, valine and isoleucine degradation suggest upregulation of these pathways in the high treatment in comparison to the low treatment animals.

**Table 3:** Genes differentially expressed in the subcutaneous adipose tissue of Holstein-Friesian dairy bulls fed on a low plane of nutrition in comparison to the high plane of nutrition and slaughtered at 18 weeks of age returned by Goseq (*P* < 0.05; False Discovery Rate < 0.05: fold change> 2.0)

Rank	KO Pathway	Pathway Name
1	01100	Metabolic pathways
2	00280	Valine, leucine and isoleucine degradation
3	00020	Citrate Cycle -carbohydrate metabolism
4	05012	Parkinson’s disease
5	00190	NADH:ubiquinone oxidoreductase
6	00640	Malonate semialdehyde pathway
7	00650	Butanoate metabolism
8	03010	Ribosome
9	00630	Glyoxylate and dicarboxylate metabolism
10	05010	Alzheimer’s disease
11	00071	Fatty acid degradation
12	00620	Pyruvate metabolism
13	00100	Steroid biosynthesis
14	03320	PPAR signaling pathway
15	00380	Tryptophan metabolism
16	05016	Huntington disease
17	00410	beta-Alanine metabolism
18	00561	Glycerolipid metabolism
19	04512	ECM-receptor interaction
20	04146	Peroxisome
21	00120	Primary bile acid biosynthesis
22	00330	Arginine and proline metabolism
23	00010	Glycolysis / Gluconeogenesis

Rank = significance relative position (1 is the most significant, 23 is the least significant) *KO* KEGG Orthology

A total of 25 networks, regarded as having biological significance were identified. Particular networks of interest including energy production, lipid metabolism and small molecule biochemistry (Network 4; Table 4) were identified as being enriched in the high compared to the low treatment. This network highlighted 28 associated molecules, 24 of which were upregulated, and 4 of which were down regulated in the high compared to the low treatment animals. Of particular interest, *ADIPOQ* (adiponectin; − 1.62 log fold change) and its receptor *ADIPOR2* (− 1.772 log fold change) and *LEP* (leptin; − 4.541 log fold change) were upregulated in the high compared to the low treatment. Using the Molecule Activity Predictor (MAP) function in IPA insulin has been predicted to be inhibited in the low compared to the high treatment (Additional file 3).

**Table 4:** Network of biological interest generated through network analysis using Ingenuity Pathway Analysis (IP A) of subcutaneous adipose tissue of Holstein-Friesian dairy bulls fed on low plane of nutrition in comparison to a high plane of nutrition, slaughtered at 18 weeks of age

Network ID	Top Functions	Effected Molecules in Network	Score	Focus Molecule
4	Energy Production, Lipid Metabolism, Small Molecule Biochemistry	*ABLIM2* ***, ACAT1, ADIPOQ, ADIPOR2, ALDOA, BCAT2, CISD1, CKB, FABP4, FITM2, GPD1, HOMER2, LEP, LGALS12, MT-ND1, MT-ND6, NDUFB5, NDUFB8, NDUFS2, NDUFV1, NNAT,*** *NR4A1, SNCA* ***, SORBS1,*** *SYNE2* ***, TUSC5***	39	28

Molecules highlighted in bold are downregulated in the low plane of nutrition in comparison to the high plane of nutrition

### Lipogenesis and adipogenesis

Animals on the low plane of nutrition had a lower of expression of *MLXIPL* (MLX interacting protein-like, also known as *ChREBP*; − 1.375 log fold change), a transcription factor that regulates lipogenesis and adipogenesis, in comparison to the high plane of nutrition. Target genes linked with adipocyte differentiation were down regulated in the low treatment in comparison to the high treatment, such as *DGAT2* (diacylglycerol acyltransferase 2; − 5.215 log fold change), *FASN* (fatty acid synthetase; − 5-184 log fold change), *ACACA* (acetyl-CoA carboxylase-α; − 3.432 log fold change), *LPL* (lipoprotein lipase; − 3.241 log fold change), *PLIN1* (Perilipin 1; − 2.279 log fold change) and *FABP4* (fatty acid-binding protein 4; − 2.273 log fold change), *INSIG-1* (insulin-induced gene 1; − 3.216 log fold change) and *GDF-10* (growth differentiation factor 10; − 2.014). Genes that act as inhibitors for adipogenesis such as *GATA2* (GATA binding protein 2; 1.151 log fold change) and *WNT2B* (Wnt family member 2B; 1.672 log fold change) were up regulated in the low plane of nutrition compared to the high.

## Discussion

This is the first study to apply next generation sequencing technology to examine the effect of early calf-hood nutrition on the transcriptomic profile of subcutaneous adipose in calves. The histological and mRNA expression data indicate much lower adiposity (number and size of adipocytes) and greater number of preadipocytes in the calves subjected to the low plane of nutrition. This was consistent with evidence for the high plane of nutrition inducing an increase in both energy production and amino acid metabolic pathways in comparison to the low treatment. The ‘energy production, lipid metabolism and small molecule biochemistry network’ was also found to be enriched in the high compared to the low group with certain genes of particular interest within this network, including *ADIPOQ*, *ADIPOR2* and *LEP* all consistent with increased adipogenesis and all upregulated in the high treatment group.

### Lipogenesis and adipogenesis

It has been postulated that rapid growth during the post weaning period in humans can cause preadipocytes to enter terminal differentiation [19]. Some transcription factors have been highlighted as regulators of lipogenesis and adipogenesis at a transcriptional level, including sterol regulatory element binding factor 1 (*SREBF1*) and MLXIPL [20]. The calves on the low plane of nutrition in our study had lower expression of *MLXIPL* in comparison to their contemporaries on the high plane of nutrition, which was supported by the complementary histology data. It has been reported that *MLXIPL* expression levels increase during preadipocyte differentiation in humans, mice and rats; however, there was a dramatic decrease in *MLXIPL* expression when cell differentiation took place in the absence of insulin [21, 22]. It is well accepted that insulin is a metabolically active hormone responsible for anabolic processes promoting growth, development, and nutrient homeostasis [23, 24]. Indeed we recorded greater plasma concentrations of insulin in the high compared with the low treatment groups at the time of slaughter (data not presented), although no differences were observed at the mRNA level. Insulin-induced gene-1 (INSIG-1) has been reported to mediate sterol regulatory element binding protein-1c (SREBP-c) which is a transcription factor involved in the regulation of the effect of insulin on lipid metabolism and adipogensis [25]. Studies have indicated that *INSIG-1* increases in the fat tissue of mice developing diet induced obesity and also in differentiating 3 T3-L1 preadipocytes [26]. This highlights the importance of insulin in lipogenesis.

In a recent study [27] the authors reported a transcriptomic analysis of adipogenesis in human adipocyte tissue and identified a variety of genes as general adipocyte markers such as *FABP4, PLIN1, LPL* and *ADIPOQ*. These genes had increased expression during adipogenic differentiation consistent with the findings of others [28]. Upregulation of *FABP4*, *FASN*, *ACACA* and *DGAT2* mRNA, which are target genes of *PPARγ,* are required for fatty acid biosynthesis and adipocyte differentiation [19, 29]. FASN is critical in the process of synthesising long chain saturated fatty acids and its function is inhibited by fasting [30]. Down regulation of *ACACA* has been reported in calorie restriction in adipose and liver tissue in pigs [31] and muscle in cattle [32]. It has been hypothesised that leptin controls adipocyte size via influencing *DGAT* expression [33]. Eventhough, no difference in *PPARγ* expression was determined in our study, downstream genes affected by *PPARγ* were downregulated in tissue from the animals on the low plane of nutrition. There was no evidence of *BMP* (bone morphogenic protein), expression being affected by plane of nutrition. However, *GDF10* which is linked to *BMP3* was found to downregulated on the low plane of nutrition. As well as a down regulation in genes that stimulate adipogenesis, in our study we found an upregulation in a key gene found to inhibit adipogenesis in the low plane of nutrition compared to the high plane of nutrition. It has been reported that GATA2 is expressed in preadipocytes and is down-regulated during terminal differentiation [34]. Studies in mouse preadipocytes show that GATA2 inhibits adipogenesis and traps cells at preadipocyte stage by direct suppression of PPARγ [34]. In addition, the histological data showing much lower adiposity (number and size of adipocytes) and greater number of preadipocytes in the animals on the low plane of nutrition corroborates the transcriptomic data aforementioned.

### Endocrine regulatory networks

Insulin plays a role in regulating the synthesis and secretion of leptin, as adipocytes cultured in the presence of insulin increased the synthesis and secretion of leptin [35, 36]. It is therefore unsurprising that IPA predicted, when applied to our data, the stimulation of insulin as well as the known upregulation of *LGALS12* (Galectin 12), *BCAT2* (Branched Chain Amino Acid Transaminase 2) and *ADIPOQ* (adiponection), which are all located upstream of *LEP* and likely resulted in *LEP* to be upregulated in the high treatment. Plasma leptin is positively correlated with body fat in growing cattle [37] where it has been shown to signal energy abundance by informing the various bodily systems, including the HPT axis, of the prevailing metabolic status and energy reserves via circulating leptin protein concentrations [12]. For example, in adult animals, studies which examined the adipose tissue transcriptome of pre-partum dairy cows have shown that cows with greater BCS had greater expression of *LEP* [38]. Consistent with this we found that calves on the high plane of nutrition in our study had > 4.5 log fold higher *LEP* expression compared with animals the low animals. Despite this, when plasma concentrations were assayed, we failed to find any difference in leptin protein concentrations between the two groups (English et al., unpublished). This is in agreement with studies in both young dairy and beef bulls [1, 2, 39]. The lack of differences in plasma leptin concentrations may be due to the minute quantity of adipose tissue laid down in calves at this stage of development and/or the relative insensitivity of protein immunoassays compared with transcript based molecular approaches.

Leptin does not directly affect GnRH neurons of the hypothalamus and knocking out of leptin receptors on the GnRH neurons does not apparently delay the onset of puberty nor indeed affect subsequent fertility [13]. However, leptin has been found to indirectly influence the timing of puberty via the regulation of the hypothalamic Kiss1 neurons’ stimulation of GnRH [10]. Leptin can also bypass the hypothalamus and act on the pituitary gland and the testes; with leptin modestly stimulating gonadotropin secretion at a pituitary level and both direct stimulatory and inhibitory actions of leptin been reported in the gonads [40]. At high leptin concentrations, it has been reported to inhibit testosterone secretion in vitro in adult rats but to have no effect in pubertal rats [41]. This is possibly due to the fact that pubertal testes produce more 5α-reduced androgens than testosterone [42]. In research carried out by Byrne et al. (2016) bulls which had experienced dietary restriction prior to 6 months of age and an increase in plane of nutrition post 6 months of age leading to an increase in leptin concentration, were older at puberty than their contemporaries offered a high plane of nutrition; highlighting the importance of early life nutrition on age at puberty.

Adiponectin functions in the regulation of lipid and glucose metabolism, insulin sensitivity as well as inflammation [43–45]. Its concentration in blood has been shown to be negatively correlated to adipocyte size [46]. Food intake inhibits *ADIPOQ* mRNA expression in WAT and therefore, inhibits adiponectin concentration in serum [47]. Globular adiponectin protein has been found to inhibit the secretion of *GnRH* in GT1-7 cells derived from mouse hypothalamic GnRH neurons via the mediation of adenosine monophosphate-activated protein kinase (AMPK; Wen et al., 2008). However, high doses of adiponectin in MA-10 mouse Leydig cells has been reported to advance progesterone production through an increase in StAR (Steroidogenic Acute Regulatory Protein) and the CYP11A1 (Cytochrome P450 Family 11 Subfamily A Member 1) steroidogenesis enzyme, suggesting that adiponectin could stimulate testosterone production from the Leydig cells [48]. It has also been shown that adiponectin reduces LH secretion directly from the gonadotropes in the anterior pituitary via AMPK [49]. Pre-pubertal children with increased body mass index (BMI), though not classified as obese, had lower adiponectin concentration in subcutaneous adipose [50]. Similarly, postpartum cows, in negative energy balance had decreased *ADIPOR1* and *ADIPOR2* abundance in subcutaneous adipose compared to antepartum cows [51] which is in agreement with our findings with calves where we found *ADIPOQ* and its receptor *ADIPOR2* were downregulated in the low compared to the high treatment groups. It has been reported that ADIPOR2 null mice demonstrated seminiferous tubules with aspermia but had normal testosterone concentration [52]. It has also been found that the abundance of *ADIPOQ*, *ADIPOR1* and *ADIPOR2* is greater in high fertility compared to that of medium and low fertility Holstein bulls [53]. There was no difference in serum concentrations of adiponectin between the two groups in the current study (English et al., unpublished), which like leptin may be due to the overall low adipose deposition in young bull calves. Using a very similar animal model and the same assays as those employed here Byrne et al. (2016); similarly observed in leptin and adiponectin concentrations during the same development as reported here. However, from 8 months on age onwards, bull calves offered a high plane of nutrition had greater leptin compared to their contemporaries on a moderate plane of nutrition (ADG c.0.65 kg/day).

### Branched chain amino acid metabolism

Branched chain amino acids (BCAAs), leucine, isoleucine and valine are essential amino acids and, unlike other amino acids which are catabolized by the liver, BCAAs are catabolized mainly in the muscle, adipose, kidney and the brain; as the liver does not contain the branched amino acid transferase enzyme (BCAT2), necessary for BCAA catabolism [54]. The isoleucine/valine degradation pathway enters the TCA cycle either directly or via an acetyl derived intermediate and in this study we found that genes involved in the valine degradation pathway encoding *BCAT2*, 2-oxoisovalerate dehydrogenase (*BCKDHB* and *DLD*), 2-methylacyl-CoA dehydrogenase (*ACADSB*), enoyl-CoA hydratase (*ECHS1*, *EHHADH*, *HADHA* and *HADHB*), 3-hydroxyisobutyryl-CoA hydrolase (*HIBCH*), methylmalonate-semialdehyde dehydrogenase (*ALDH6A1*) and (S)-3-amino-2-methylpropionate transaminase (*ABAT*) were all down regulated in the low in contrast to the high treatment. Similarly, within the isoleucine degradation pathway, genes encoding for branched-chain-amino-acid transaminase (*BCAT2*), branched-chain α-keto acid dehydrogenase complex (*DLD*), 2-methylacyl-CoA dehydrogenase (*ACADSB*), enoyl-CoA hydratase (*ECHS1, EHHADH, HADHA* and *HADHB*), 3-hydroxy-2-methybutyryl-CoA dehydrogenase (*HSD17B10*) and acetyl-CoA C-acetyltransferase (*ACAT1*) were down regulated in the low compared to the high treatment. Expression of genes involved in branched chain amino acids metabolism were upregulated during 3 T3-L1 adipocyte differentiation [55, 56] suggesting that BCAA are involved in fatty acid synthesis [57]. It has also been shown that BCAA, especially leucine, systemic concentrations increase leptin secretion in vitro in murine [58] and decreases food intake in vitro in rats [59]. Systemic concentrations of branched chain amino acid are elevated in response to over nutrition in children and adolescents [60]. Therefore, it is reasonable to suggest that the evidence for a decline in BCAA degradation may be a result of the moderate restriction in dietary allowance experienced by our low plane of nutrition calves. The BCAA catabolic by-products mentioned in this study are intended for integration into the TCA cycle and therefore, any down regulation of the BCAA degradation pathway would cause a decrease in mitochondrial respiration in existing adipocytes [61].

### Energy production and mitochondrial dysfunction

Mitochondria are key organelles in cellular energetics as they catabolise carbohydrates, lipids and proteins to synthesise ATP and metabolites for not only growth but also adipocyte differentiation and maturation [62]. There are four stages involved in aerobic respiration. Glycolysis occurs in the cytoplasm and the remaining steps, pyruvate oxidation, the TCA cycle and electron transport chain and chemiosmosis (as employed in oxidative phosphorylation) take place within the inner membrane of the mitochondria [63]. The two pyruvate molecules, the products of glycolysis, enter the mitochondrion and are converted to acetyl coenzyme A (acetyl CoA). Acetyl CoA enters the TCA cycle which drives the cycle to produce NADH, ATP and FADH_2_. Genes encoding for citrate synthase (*CS*) namely, aconitase (*ACO1* and *ACO2*), isocitrate dehydrogenase (*IDH3B*), α-ketoglutarate dehydrogenase (*OGDH* and *DLD*), succinyl-CoA synthetase (*SUCLG1*), fumarase (*FH*) and malate dehydrogenase (*MDH1*), were all found to be down regulated in the low compared to the high treatment. NADH and FADH_2_ donate electrons to the electron transport chain, which powers ATP synthesis via oxidative phosphorylation. Genes associated with NADH dehydrogenase Complex I, succinate dehydrogenase Complex II, ubiquinol-cyt c oxidoreductase Complex III, cytochrome c oxidase Complex IV and ATP synthase Complex V were down regulated in the low plane of nutrition compared to the high plane of nutrition (Table 5). It has been reported that there is an increase in mRNA expression of nucleus-encoded mitochondrial genes for enzymes such as pyruvate carboxylase and pyruvate dehydrogenase complex during differentiating 3 T3-L1 adipocytes [64]. Pyruvate carboxylase has been found to be overexpressed upon conversion of preadipocytes to adipocytes [65]. This is consistent with the results of our study where the high plane of nutrition calves were at a more advanced stage of adipocyte differentiation, at tissue recovery, as indicated by the significant number of adipocytes following histological investigation when compared with their lower nutrition counterparts.

**Table 5:** Oxidative Phosphorylation genes differentially expressed in the subcutaneous adipose tissue of the Holstein-Friesian dairy bulls feed on a low plane of nutrition in comparison to the high plane of nutrition and slaughtered at 18 weeks of age

Symbol	Gene Name	Log Fold Change
Complex I		
NDUFA4	NDUFA4, mitochondrial complex associated	−1.472
NDUFA5	NADH:ubiquinone oxidoreductase subunit A5	−1.117
NDUFA8	NADH:ubiquinone oxidoreductase subunit A8	−1.134
NDUFA9	NADH:ubiquinone oxidoreductase subunit A9	−1.202
NDUFA12	NADH:ubiquinone oxidoreductase subunit A12	−1.221
NDUFB3	NADH:ubiquinone oxidoreductase subunit B3	−1.401
NDUFB5	NADH:ubiquinone oxidoreductase subunit B5	−1.269
NDUFB8	NADH:ubiquinone oxidoreductase subunit B8	−1.137
NDUFS1	NADH:ubiquinone oxidoreductase core subunit S1	−1.711
NDUFS2	NADH:ubiquinone oxidoreductase core subunit S2	−1.464
NDUFS4	NADH:ubiquinone oxidoreductase subunit S4	−1.196
NDUFS7	NADH:ubiquinone oxidoreductase core subunit S7	−1.494
NDUFS8	NADH:ubiquinone oxidoreductase core subunit S8	−1.328
NDUFV1	NADH:ubiquinone oxidoreductase core subunit V1	−1.703
MT-ND1	NADH dehydrogenase, subunit 1 (complex I)	−1.058
MT-ND2	MTND2	−1.182
MT-ND3	NADH dehydrogenase, subunit 3 (complex I)	−1.035
MT-ND4	NADH dehydrogenase, subunit 4 (complex I)	−1.522
MT-ND5	NADH dehydrogenase, subunit 5 (complex I)	−1.848
MT-ND4L	NADH dehydrogenase, subunit 4 L (complex I)	−1.552
Complex III		
UQCR10	ubiquinol-cytochrome c reductase, complex III subunit X	−1.629
UQCR11	ubiquinol-cytochrome c reductase, complex III subunit XI	−1.217
UQCRB	ubiquinol-cytochrome c reductase binding protein	−1.498
UQCRC1	ubiquinol-cytochrome c reductase core protein I	−1.479
UQCRC2	ubiquinol-cytochrome c reductase core protein II	−1.532
MT-CYB	cytochrome b	−1.647
Complex IV		
COX5A	cytochrome c oxidase subunit 5A	−1.567
COX5B	cytochrome c oxidase subunit 5B	−1.566
COX6B1	cytochrome c oxidase subunit 6B1	−1.049
COX6C	cytochrome c oxidase subunit VIc	−1.115
COX7A2	cytochrome c oxidase subunit 7A2	−1.061
CYB5A	cytochrome b5 type A	−1.559
CYC1	cytochrome c1	−1.072
CYCS	cytochrome c, somatic	−1.169
MT-CO1	cytochrome c oxidase subunit I	−1.695
MT-CO2	cytochrome c oxidase subunit II	−1.39
MT-CO3	cytochrome c oxidase III	−1.161
Complex V		
ATP5A1	ATP synthase, H+ transporting, mitochondrial F1 complex, alpha subunit 1, cardiac muscle	−1.339
ATP5B	ATP synthase, H+ transporting, mitochondrial F1 complex, beta polypeptide	−1.205
ATP5C1	ATP synthase, H+ transporting, mitochondrial F1 complex, gamma polypeptide 1	−1.186
ATP5D	ATP synthase, H+ transporting, mitochondrial F1 complex, delta subunit	−1.246
ATP5G1	ATP synthase, H+ transporting, mitochondrial Fo complex subunit C1 (subunit 9)	−1.183
ATP5G3	ATP synthase, H+ transporting, mitochondrial Fo complex subunit C3 (subunit 9)	−1.548
ATP5J2	ATP synthase, H+ transporting, mitochondrial Fo complex subunit F2	−1.205
ATP5O	ATP synthase, H+ transporting, mitochondrial F1 complex, O subunit	−1.073
MT-ATP6	ATP synthase F0 subunit 6	−1.22

## Conclusion

This study clearly demonstrates that the prevailing plane of nutrition of bull calves during the first 18 weeks of life can have a significant effect on the transcriptional and morphological functionality of subcutaneous adipose tissue. A high plane of nutrition during early calf hood has been shown to positively affect adipokines, leptin and adiponectin whose functions include signalling metabolic status to the reproductive system. The data generated in this study broaden our knowledge base around the potential molecular processes underlying the biological cross-talk between peripheral tissues that may be metabolic mediators of important events such as timing of puberty onset etc. Furthermore, global differential gene expression patterns provide data which may have implications for the selection of robust biomarkers to identify animals with superior genetic potential for early pubertal onset, greater carcass adiposity and other economically important traits, shown to be latently affected by early life plane of nutrition.

## Additional files

Additional file 1: Molecular and cellular function of differentially expressed genes of the subcutaneous adipose tissue of Holstein-Friesian dairy bulls fed on a low versus high plane of nutrition and slaughtered at 18 weeks of age. The bars indicate the likelihood [−log (*P*-value)] that the specific molecular and cellular function was affected by a high plane of nutrition. The threshold line in the bar chart represents a *p*-value of 0.05. (PDF 1520 kb)

Additional file 2: Biochemical pathway significantly enriched in the subcutaneous adipose tissue of Holstein-Friesian dairy bulls fed on a low plane of nutrition in comparison to a high plane of nutrition, slaughtered at 18 weeks of age. Greens bars represent genes down regulated and red bars up regulated genes as percentages of the overall number of genes in each pathway. The significance of each pathway is represented by the orange line describing –log(p-value). The numbers along the right hand side indicate the number of genes in total involved in each pathway. The *P*-value is calculated by the number of genes from our dataset of differentially expressed genes that participate in a particular pathway and dividing it by the total number of genes in the Canonical Pathway in Ingenuity Pathways Analysis (IPA) analysis. (PDF 4096 kb)

Additional file 3: Genes differentially expressed in the subcutaneous adipose of Holstein-Friesian dairy bulls fed on low plane of nutrition in comparison to high plane of nutrition, slaughtered at 18 weeks of age. Genes outlined by the Molecule Activity Predictor function in IPA. (XLS 106 kb)

## Abbreviations

ACACA: Acetyl-CoA carboxylase-α
ADIPOQ: Adiponectin
AMPK: Adenosine monophosphate-activated protein kinase
BCAA: Branched chain amino acids
BCAT2: Branched chain amino acid transaminase 2
BCS: Body conditioning score
BMI: Body mass index
BMP: Bone morphogenic bone
bp: Base pairs
CYP11A1: Cytochrome P450 Family 11 Subfamily A Member 1
DEG: Differentially expressed genes
DGAT2: Diacylglycerol acyltransferase 2
FABP4: Fatty acid-binding protein 4
FASN: Fatty acid synthetase
FDR: False discovery rate
GATA2: GATA binding protein 2
GDF-10: Growth differentiation factor 10
GnRH: Gonadotropin releasing hormone
H&E: Haematoxylin and eosin
HPT: Hypothalamic-pituitary-testicular
INSIG-1: Insulin-induced gene 1
IPA: Ingenuity pathway analysis
KEGG: Kyoto Encyclopaedia of genes and genomes
KISS1: Kisspeptin
LEP: Leptin
LGALS12: Galectin 12
LH: Luteinising hormone
LPL: Lipoprotein lipase
MAP: Molecule activity predictor
MDS: Multi-dimensional scaling
MLXIPL/ChREBP: MLX interacting protein-like
PLIN1: Perilipin 1
RIN: RNA integrity number
RNAseq: RNA sequencing
SREBF1: Sterol regulatory element binding factor 1
SREBP-c: Sterol regulatory element binding protein-1c
STAR: Spliced transcripts alignment to a reference
StAR: Steroidogenic acute regulatory protein
TCA: Tricarboxylic acid
WAT: White adipose tissue
WNT2B: Wnt family member 2B
