# Supplementary figures and images for: Effect of early calf-hood nutrition on the transcriptomic profile of subcutaneous adipose tissue in Holstein-Friesian bulls
Source: BMC Genomics. 2018 Apr 24;19:281. doi: 10.1186/s12864-018-4681-2 (PMC5916831; doi:10.1186/s12864-018-4681-2)

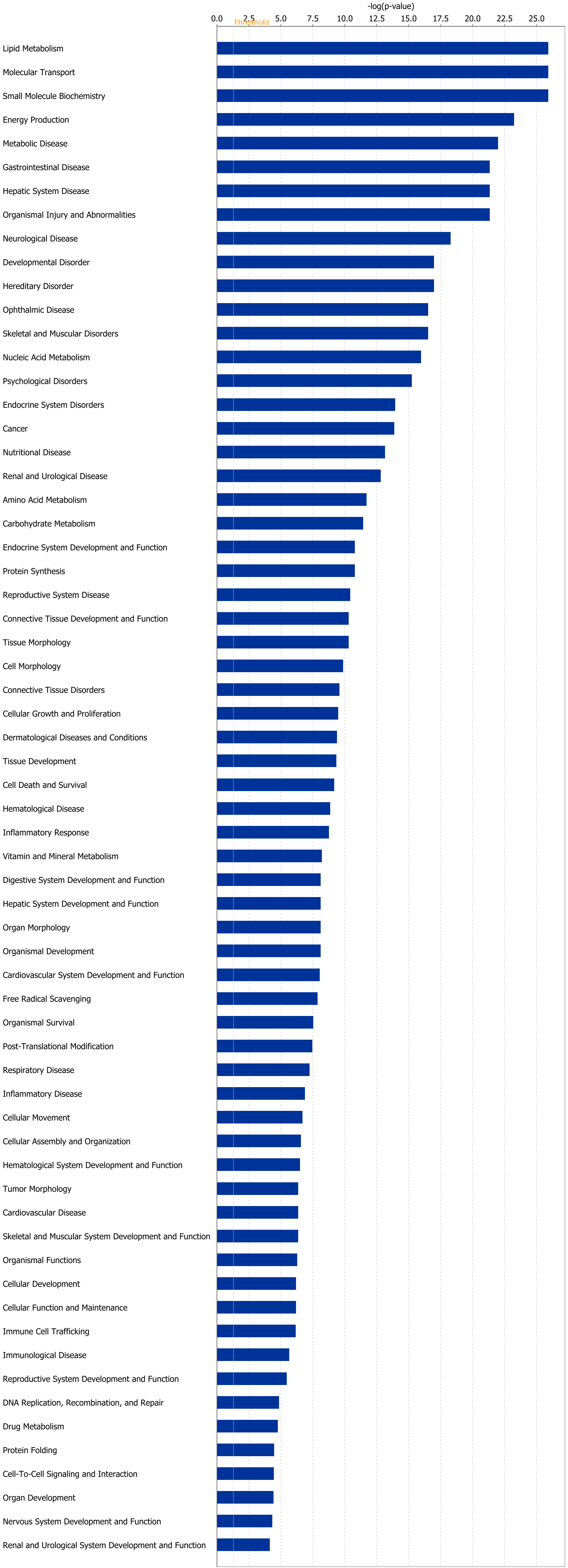

Supplement: Supplementary file 1 — Molecular and cellular function of differentially expressed genes of the subcutaneous adipose tissue of Holstein-Friesian dairy bulls fed on a low versus high plane of nutrition and slaughtered at 18 weeks of age. The bars indicate the likelihood [−log (P-value)] that the specific molecular and cellular function was affected by a high plane of nutrition. The threshold line in the bar chart represents a p-value of 0.05. (PDF 1520 kb) [file 12864_2018_4681_MOESM1_ESM.pdf]

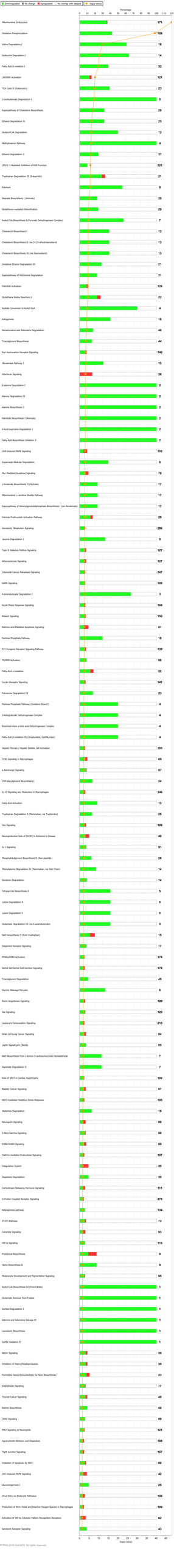

Supplement: Supplementary file 2 — Biochemical pathway significantly enriched in the subcutaneous adipose tissue of Holstein-Friesian dairy bulls fed on a low plane of nutrition in comparison to a high plane of nutrition, slaughtered at 18 weeks of age. Greens bars represent genes down regulated and red bars up regulated genes as percentages of the overall number of genes in each pathway. The significance of each pathway is represented by the orange line describing –log(p-value). The numbers along the right hand side indicate the number of genes in total involved in each pathway. The P-value is calculated by the number of genes from our dataset of differentially expressed genes that participate in a particular pathway and dividing it by the total number of genes in the Canonical Pathway in Ingenuity Pathways Analysis (IPA) analysis. (PDF 4096 kb) [file 12864_2018_4681_MOESM2_ESM.pdf]
